# The Use and Effectiveness of Different Emergency Contraception Methods Among Adolescent Girls and Young Women in a Greek Clinic: A Cross-Sectional, Comparative, Observational Study

**DOI:** 10.3390/clinpract15110212

**Published:** 2025-11-18

**Authors:** Athanasia Chatzilazarou, Christina Pagkaki, Anastasia Bothou, Vasiliki Kourti, Dimitrios Lamprinos, Nektaria Kritsotaki, Efthymios Oikonomou, Nikolaos Machairiotis, Angeliki Gerede, Nikoletta Koutlaki, Panagiotis Tsikouras

**Affiliations:** 1 Department of Obstetrics and Gynecology, Democritus University of Thrace, 68100 Alexandroupolis, Greece; athanasiahatzilazarou@gmail.com (A.C.);; 2 Department of Midwifery, School of Health Sciences, University of West Attica (UNIWA), 12243 Egaleo, Greece; 3 Laboratory of Hygiene and Environmental Protection, Department of Medical Micobiology-Immunology, Democritus University of Thrace, 68100 Alexandroupolis, Greece; 4 Department of Obstetrics and Gynecology, National and Kapodistrian University of Athens, Atticon, 12462 Athens, Greece

**Keywords:** emergency contraception, different populations, contraception methods, IUD, ulipristal acetate, levonorgestrel

## Abstract

**Background:** Emergency contraception (EC), also known as postcoital contraception, is a method used to prevent an unintended pregnancy following unprotected or inadequately protected sexual intercourse. The available options include emergency contraceptive pills or the insertion of an intrauterine device (IUD). Emergency contraception pills contain either levonorgestrel (a single 1.5 mg dose, effective within 72 h) or ulipristal acetate (a single 30 mg dose, effective within 120 h), both of which are most effective when taken as soon as possible after unprotected intercourse. Another highly effective option is the insertion of a copper or levonorgestrel-releasing intrauterine device, although IUDs are not registered for EC use in all countries. The aims of this cross-sectional, comparative, observational study were to collect data on the emergency contraception methods used by adolescent girls and young women to examine their association with various factors, such as religious beliefs, and to evaluate the effectiveness of different emergency contraception methods, including hormonal options and intrauterine devices. **Methods**: Data were collected from 240 women who attended our Family Planning Clinic using a structured questionnaire that included items on their demographic characteristics, religious beliefs, medical history, lifestyle factors, contraceptive use and side effects, prior use of emergency contraception, method selected, and reasons for seeking emergency contraception. Descriptive statistics were used to summarize the data, comparisons between religious groups were conducted using chi-square tests, and factors related to the timing of emergency contraceptive use were investigated using multinomial logistic regression analysis. **Results:** Most of the reasons for emergency contraception use did not differ significantly between Christian and Muslim participants. However, Christians were significantly more likely to use emergency contraception due to missed contraceptive doses (20.9% vs. 6.7%, *p* = 0.004) or the failure to take a progesterone-only pill (19.1% vs. 3.3%, *p* = 0.001). Levonorgestrel was the most frequently used method in both groups (48.9% of Christians vs. 60% of Muslims, *p* = 0.132), followed by ulipristal acetate (30.9% vs. 40%, *p* = 0.180). Notably, 18.5% of Christian participants used an intrauterine device (IUD) for emergency contraception, while no Muslim participants reported IUD use (*p* < 0.001), indicating a significant difference potentially influenced by cultural or religious factors. **Conclusions:** Both religious and individual sociodemographic factors affect not only the choice of emergency contraception but also the urgency with which the emergency contraception is used. Interventions aimed at improving contraception education, addressing partner-related challenges, and promoting timely access could improve reproductive health outcomes.

## 1. Introduction

Unintended pregnancies are a major worldwide public health concern that highlight the need for accessible and effective contraception methods [1]. Approximately 61% of unwanted pregnancies are resolved through abortion, which can cause significant morbidity, especially when performed in unsafe conditions [2]. Despite significant advancements and the widespread promotion of various effective contraceptive methods in recent decades, no current method offers 100% protection against pregnancy. Emergency contraception (EC), also known as postcoital contraception, serves as a critical backup option following unprotected sexual intercourse (UPSI) or contraceptive failure to prevent unintended pregnancies. EC is recommended in situations such as unplanned intercourse without contraception, the suspected failure of regular contraceptive methods (e.g., missed pills, condom breakage or slippage, or intrauterine device displacement), and sexual assault [3].

The primary oral EC options consist of a progestin-only pill containing 1.5 mg of levonorgestrel (LNG) and a pill containing 30 mg of ulipristal acetate (UA). Access to UA varies from over-the-counter (OTC) access in some countries to prescription-only access in others [4,5]. A further option that has been widely studied and applied globally is the use of intrauterine devices (IUDs), particularly copper IUDs.

Although both LNG and UPA are highly effective at preventing pregnancy if taken timely, the copper IUD appears to be the most effective emergency contraceptive method, with a failure rate of less than 0.1% [6,7]. The efficacy of UA is also higher than that of LNG, especially at the end of the 120 h postintercourse window and in women with higher body mass indexes (BMIs) [8]. The choice of a particular method is determined by its availability, knowledge of the methods, the prescribing patterns, and sociocultural factors, which can vary between countries and different health systems [9]. In Greece, for instance, the use of over-the-counter LNG pills remains the rule for EC, while IUD use is not common due to the need for medical intervention and the lack of appropriate information concerning its use, especially among nulliparous women. For example, in a 2021 national survey of Greek tertiary students, fewer than 5% had ever considered using an IUD as an EC method, whereas 46% reported at least one episode of LNG pill use. This uptake pattern highlights a large evidence–practice gap that may be putting Greek women at risk for preventable EC failures [10].

The early onset of sexual activity in adolescent girls and the increasing number of sexual partners emphasize the need to incorporate sexual education into public health systems to delay sexual activity initiation, increase contraceptive use, and decrease high-risk sexual behaviors [11,12].

In this study, we sought to examine the contraceptive practices among adolescent females and young women in Thrace, Greece, across an eleven-year span, emphasizing the correlation between religious identification, socioeconomic variables, and the decision making process regarding the selection and timing of emergency contraception utilization. Our hypothesis posited that both religious identification and socioeconomic status impact the chosen method (hormonal compared with IUD) and the immediacy associated with emergency contraception use.

As far as we are aware, this is the first Greek study to compare the EC use behaviors between Christian and Muslim women and examine the sociocultural and behavioral determinants. By filling this niche, this study addresses the utilization of regionally specific information to produce targeted teaching and clinical interventions to improve reproductive health outcomes.

## 2. Methods

This cross-sectional, comparative, observational study was conducted to evaluate the use and effectiveness of different emergency contraception (EC) methods—levonorgestrel, ulipristal acetate, and the intrauterine device (IUD)—and to identify factors influencing their selection and timing among women of reproductive age.

A total of 240 participants were enrolled from the Family Planning Clinic of the Department of Obstetrics and Gynecology at Democritus University of Thrace, Greece. The sample comprised 110 Christian women, 90 Muslim women, and 40 Christian women who used IUDs for emergency contraception. The IUD group was analyzed separately due to the distinction of the method and because no Muslim participants reported IUD use, reflecting the potential influence of cultural or religious norms on the method selection.

Ethical approval for this study was obtained from the Ethics Committee of the Democritus University of Thrace (Reference No. 45183/25.10.2021). All participants provided written informed consent prior to their participation.

Eligible participants were sexually active adolescent or early-reproductive-age women who presented to the Family Planning Clinic after unprotected sexual intercourse. Unprotected intercourse was defined as any of the following: failure to use contraception, condom rupture or leakage, displacement of a diaphragm or cervical cap, missed oral contraceptive pills (≥3 pills or during the first week of the cycle), failure to take a progestogen-only pill, use of the withdrawal method, or cases of sexual abuse without reliable contraception.

Data were collected using a structured, in-house-developed questionnaire created by the research team specifically for this study. The questionnaire was reviewed and approved by the Scientific Committee of the University Hospital of Alexandroupolis to ensure content relevance, clarity, and ethical compliance, and it was also pilot-tested on 20 women before its use in the main study.

A small proportion of participants did not provide complete information on all the variables. These missing data were not imputed or statistically adjusted for in this study and may slightly limit the generalizability of the subgroup comparisons.

The questionnaire included items on demographic characteristics, prior medical history (e.g., allergic reactions, bleeding abnormalities, hypertension, metabolic or malignant diseases), lifestyle habits (smoking), and contraception use and related side effects. Participants also reported whether they had used emergency contraception in the past, the method chosen, and the reason for seeking emergency contraception at the time of their appointment.

Statistical Analysis

Descriptive statistics (frequencies and percentages) were used to portray participant characteristics. Chi-square tests were conducted to compare the groups between Christians and Muslims. Multinomial logistic regression analysis was conducted to examine the predictors of the EC use timing, and the EC use timing intervals (“within 12 h,” “within 72 h,” and “within 96 h,” used as the reference category) were compared. Independent variables included residence (urban vs. rural), age at first sexual intercourse (<16 years vs. 16–21 years), first contraceptive method (condom, pill, IUD, withdrawal, or none), and reasons for not using contraception (lack of knowledge, partner opposition, or other).

Categorical predictors were entered as factors, and reference categories were assigned for interpretability. Odds ratios (ORs) and 95% confidence intervals (CIs) were calculated to assess the direction and magnitude of the associations. A *p*-value < 0.05 was considered statistically significant. Analyses were conducted using SPSS software version 23.

## 3. Results

**Figure 1:**
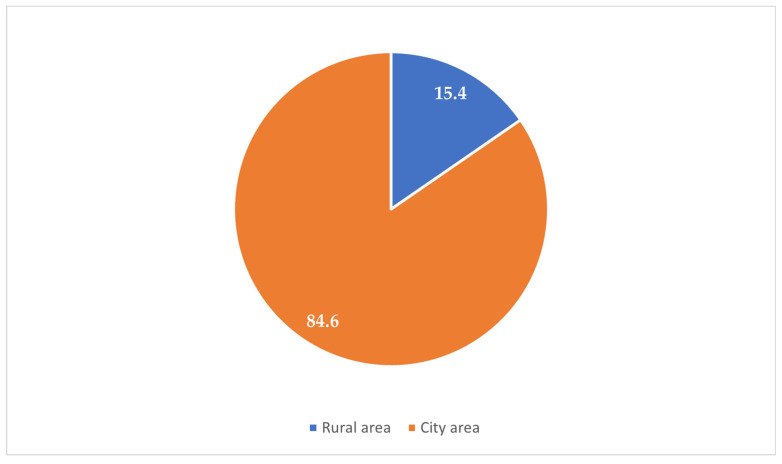
Rural vs. urban residence.

**Figure 2:**
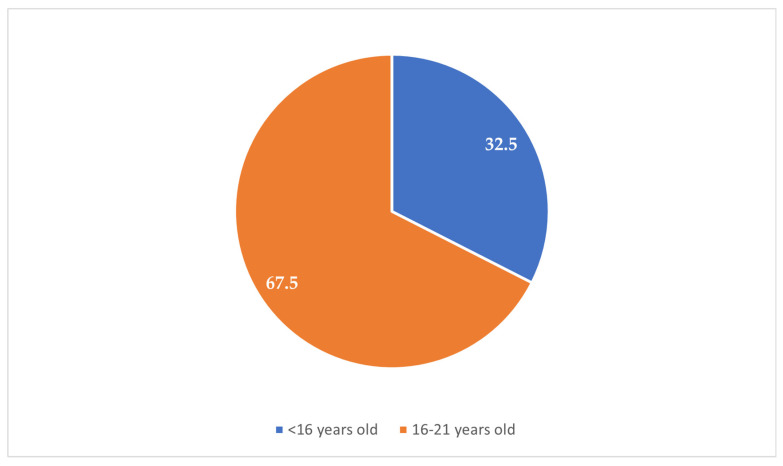
Age at first sexual intercourse.

**Figure 3:**
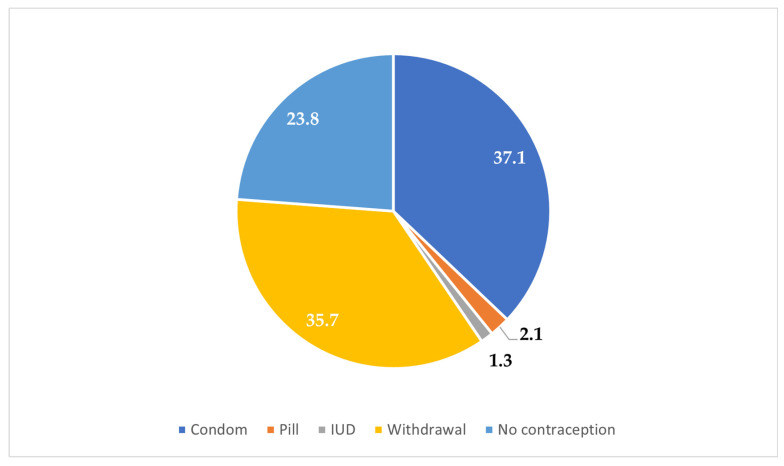
Contraception used during first sexual intercourse.

**Figure 4:**
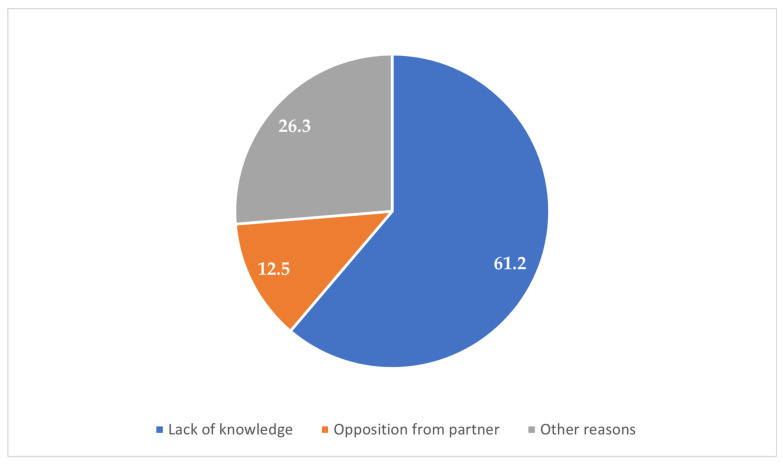
Reasons for not using contraception.

**Figure 5:**
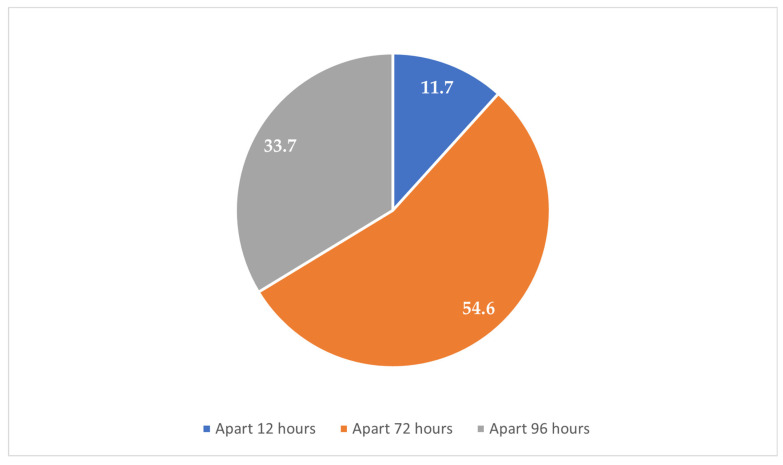
Timing of emergency contraception use.

The majority of the participants (84.6%) resided in urban areas, while 15.4% were from rural zones (Figure 1). Most participants (67.5%) reported experiencing their first sexual intercourse between the ages of 16 and 21 years, whereas 32.5% reported that it occurred before the age of 16 years (Figure 2). Condoms were the most commonly reported (37.1%) method used during the first sexual encounter, followed by withdrawal (35.7%). A notable portion (23.8%) did not use any contraception at all, and a small number reported using the contraceptive pill (2.1%) or an IUD (1.3%) (Figure 3). Among those who did not use contraception, the most frequently cited reason was a lack of knowledge (61.2%), followed by partner opposition (12.5%) and other personal or contextual reasons (26.3%) (Figure 4). Emergency contraception was used within 12 h by 11.7% of participants, within 72 h by 54.6%, and within 96 h by 33.7% (Figure 5).

**Figure 6:**
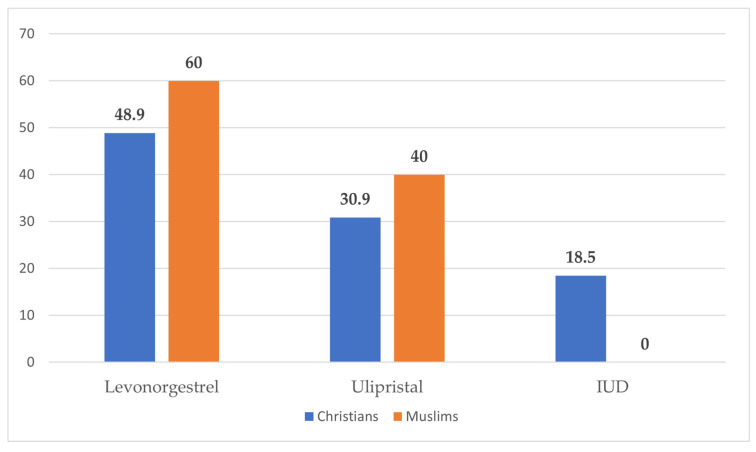
Use of emergency contraception methods by religion.

A total of 240 women participated in this study, including 110 Christians, 90 Muslims, and 40 participants with IUDs, who were all Christians (Figure 6). Levonorgestrel was the most commonly used method overall, reported by 48.9% of Christian women and 60% of Muslim women. Ulipristal acetate was the second most frequently chosen method, used by 30.9% of Christians and 40% of Muslims. The most interesting finding regarding the use of the intrauterine device (IUD) was that 18.5% of Christian participants reported IUD use, while none of the Muslim participants did (Figure 1).

Among the 110 Christian women, 65.1% reported being smokers. Abnormal uterine bleeding was reported by 90.9%, while allergic reactions (2.7%) were rare. During their first intercourse, 35.5% of participants used condoms, 34.5% used withdrawal, and 27.3% did not use contraception. The use of oral contraceptive pills was reported by 2.7%. Among those who used no method, the main causes were a lack of knowledge (52.5%), partner opposition (12.5%), and other factors (35%). Regarding regular contraceptive use, 28.2% of participants reported not using any method, 33.6% used condoms, and 38.2% reported using oral contraceptive pills. As far as information sources are concerned, only 22% had received guidance from health professionals. The blood group distribution was consistent across types and comparable with that of Muslims (*p* = 0.900).

Among the 90 Muslim participants, 61.1% were smokers (*p* = 0.557, compared with Christians). Abnormal bleeding was reported by 96.7% (*p* = 0.100, compared with Christians), while allergic reactions occurred in 3.3% (*p* = 0.803, compared with Christians). During the first intercourse, 40% used condoms, 37.8% used withdrawal, and 21.1% used no method. Oral contraceptive use was rare (1.1%), and no participant reported IUD use. Among those who did not use contraception, a lack of knowledge was the main reason (73.7%), which was reported significantly more frequently among Muslims than among Christians (*p* = 0.004). Partner opposition (13.2%) and other contextual reasons (13.2%) were mentioned less often. Regarding routine contraception, 21.1% used no method, 41.1% used condoms, and 37.8% used pills (*p* = 0.419). Information from health professionals was reported by 24.7% (*p* = 0.654, compared with Christians). No significant differences were found in the blood group distribution (*p* = 0.900, compared with Christians).

The reasons for emergency contraception use did not differ significantly between Christians and Muslims and were as follows: contraceptive failure (14.5% vs. 14.4%, *p* = 0.984), condom rupture (10.9% vs. 8.9%, *p* = 0.636), method displacement (5.5% vs. 3.3%, *p* = 0.472), use of withdrawal (12.7% vs. 11.1%, *p* = 0.726), and no method used (27.3% vs. 26.7%, *p* = 0.923). However, Christians were significantly more likely to seek emergency contraception due to missed contraception doses (20.9% vs. 6.7%, *p* = 0.004) and the failure to take a progesterone-only pill (19.1% vs. 3.3%, *p* = 0.001). Levonorgestrel was the most commonly used method in both groups (48.9% of Christians vs. 60% of Muslims, *p* = 0.132), followed by ulipristal acetate (30.9% vs. 40%, *p* = 0.180). Notably, 18.5% of Christian participants used IUDs as emergency contraception, while no Muslim participant reported IUD use (*p* < 0.001), suggesting a significant cultural or religious difference in the method selection.

### Multivariate Logistic Regression Analysis

Multivariate logistic regression analysis was used to investigate the factors influencing the timing of emergency contraception use, comparing the following time intervals of use: within 12 h, within 72 h, and within 96 h after unprotected intercourse (Table 1). Several predictors were found to be statistically significant. Participants residing in rural areas were over four times more likely to use emergency contraception within 12 h compared with those in urban areas (OR = 4.23, 95% CI [1.20, 14.97], *p* = 0.025), compared with 96 h. Similarly, those who reported using withdrawal as their first method of contraception were significantly more likely to act within 12 h (OR = 4.61, 95% CI [1.24, 17.17], *p* = 0.023).

Moreover, the reason for not using contraception significantly predicted the emergency contraception timing. Participants who reported a lack of knowledge about contraceptive methods were over four times more likely to seek emergency contraception within 12 h compared with those who delayed until 96 h (OR = 4.49, 95% CI [1.30–15.52], *p* = 0.017). Likewise, those who reported partner opposition were nearly 23 times more likely to act within 12 h (OR = 22.82, 95% CI [2.73–190.42], *p* = 0.004). When comparing emergency contraception use between within 72 h and within 96 h, the same predictors remained significant. Participants who mentioned partner opposition (OR = 4.36, *p* = 0.015), a lack of knowledge (OR = 2.36, *p* = 0.015), or other personal reasons (OR = 5.03, *p* = 0.007) were all significantly more likely to use emergency contraception earlier. While some odds ratios were high, due to the broad CI, the effect is imprecise and should be interpreted carefully.

## 4. Discussion

### 4.1. Overview of Findings

This study offers an overview of emergency contraception (EC) use among women with different religious backgrounds in Northern Greece, providing insights into the effectiveness, accessibility, and timing of the use of hormonal contraceptives and intrauterine devices (IUDs). The findings highlight the personal and social factors that influence contraception choices and decision making. Christians and Muslims shared similar demographic profiles and contraceptive behaviors, yet their method choices differed significantly: a total of 18.5% of Christian participants used IUDs for EC, while no Muslims did, elucidating how cultural and religious factors affect the choice of method used. This difference reflects global evidence showing that institutional or religious restrictions can lead to specific refusals of or preferences for long-acting reversible contraception, such as IUDs [11].

Furthermore, the EC timing was strongly influenced by variables such as urban residence, previous use of the withdrawal method as contraception, ignorance of a contraceptive method, and opposition between partners. These findings highlight that beyond medical accessibility, sociocultural and interpersonal dynamics are crucial determinants of timely EC utilization.

### 4.2. Cultural and Religious Influences

Although our observational study found that participants who reported “lack of knowledge” or “partner opposition” were notably more likely to use EC within 12 h, this is in contrast to evidence from Ghana. A qualitative investigation of unmarried women in Accra showed that prevalent misinformation and social stigma regarding EC—especially concerns regarding the safety, side effects, and moral limits—frequently resulted in delayed or incorrect use instead of early use. This divergence indicates how, in contrast to our findings, principal barriers such as inadequate knowledge and disapproval by partners in the global setting—particularly in urban Ghana—work to inhibit timely EC use, emphasizing the role of cultural and ethical factors in such relations [12].

Additionally, regular contraceptive use patterns demonstrated considerable gaps: almost one-third of the participants were non-users, and a significant difference was noted in knowledge-related reasons for non-use among religious groups. The same trends have been reported in sub-Saharan Africa, where not knowing about ECs and early sexual life greatly influenced EC use [13,14]. This alignment underscores the essential need for custom sexual education programs that respect cultural diversity.

### 4.3. Greek-Specific Insights

In Greece, contraceptive use is still defined by a heavy dependence on traditional or short-acting methods, while the use of long-acting reversible contraception (LARC) in the form of intrauterine devices (IUDs) remains much lower. In a nationwide survey of Greek women, the most frequently used methods by the respondents were male condoms (33.9%) and withdrawal (28.8%), while IUDs were adopted by only 3.6% of the interviewed females [15]. Another review also established that the prevalence of the use of modern contraceptives in Greece remains among the lowest in Europe, while the incidence of induced abortion remains very high, in spite of gaps in the availability of contraceptive education [16].

Recent research among university students confirms these trends: the use of emergency contraception was common in a multicenter study of 3624 Greek female university students aged 18–26, especially among older respondents, smokers, and those reporting multiple sexual partners [17]. In a smaller Northern Greek study among dental students, only 9% correctly identified the IUD as the most effective form of contraception, reflecting poor awareness of its inclusion among both regular and emergency contraception methods [18]. In addition, national advice technically acknowledges IUD insertion as an emergency method; however, in practice, it is uncommon owing to poor awareness among the population and among providers, who are hesitant [19].

### 4.4. Access and Health Professional Involvement

Obtaining EC with professional help is still uncommon, as only 23% of people seek advice from healthcare experts. New studies on putting EC into practice have shown that doctors and healthcare systems still face obstacles when it comes to providing IUDs for EC, even in places with official guidelines [20].

Young adult and adolescent women face a particular challenge in identifying acceptable, safe, and effective contraception. Gaps remain despite technological innovation. For example, in 2019, just over 30% of the 300 million women aged 15–19 were using any method of contraception, while an estimated 15 million failed to use a family planning method. Τhe global percentage of adolescents whose demand was met by the use of modern methods was only 59%, and this percentage was substantially less than that for adult women (76%) [21,22]. This deficit arises not only from youth-specific developmental factors but also from the lack of an ideal universal contraceptive method as per individual needs.

### 4.5. Adolescent Sexual Health and Education

Nearly one-half of the pregnancies in 15–19-year-old girls are unintended, and some occasionally end in unsafe abortions, with severe health and societal repercussions. This fact is further compounded by ongoing obstacles: numerous adolescents receive inadequate sexual education and have limited access to adolescent-friendly health services, and their autonomy is restrained regarding their choice of contraception [23]. Considering that there are 1.2 billion teenagers globally, addressing these needs is essential to reduce unplanned pregnancies and enhance reproductive health outcomes.

Beyond pregnancy, teenage sexual activity carries hazards, such as STIs, which continue to be a major public health issue for this age group [24]. Three essential pillars support optimal adolescent sexual health: acknowledging sexual rights; providing thorough sex education and counseling; and guaranteeing private, high-quality services [25,26,27,28,29]. These factors must be addressed collectively to enable a comprehensive approach to contraceptive treatment that meets the reproductive and sexual needs of young people [30,31]. Research has demonstrated that educating teenagers about the risks, advantages, and uncertainties regarding contraception in an approachable and straightforward manner helps them make well-informed decisions. Approximately 15% of teenage girls in affluent nations have their sexual debut before the average age of 17 years [32].

A holistic strategy to adolescent sexual well-being—one that pairs high-quality school-based sex education with available contraceptive services—has been exceedingly successful at preventing unwanted pregnancies and abortions. For example, Finland’s investment in compulsory sex education since the 1970s and its implementation of free contraception programs resulted in a 66% decline in teenage abortions from 2000 to 2023 [33,34]. Therefore, sex education appears to play a significant role in raising contraception awareness among teenagers.

### 4.6. Contraceptive Methods Worldwide

Contraceptive methods for teens are the same as those for adults and consist of condoms, withdrawal, combined oral contraceptives, progestin-only pills (mini-pills), intrauterine devices (IUDs), vaginal rings, diaphragms, spermicides, injectables, and emergency contraception after unprotected sex. While every contraceptive method has its advantages, drawbacks, and proven effectiveness, oral contraceptives and condoms remain the most commonly used options among adolescents [23]. Safe administration hinges on confirming ovulation and verifying contraceptive suitability. Crucially, adherence to the chosen method significantly influences its success at this sensitive age [35,36].

The increased use of methods such as medroxyprogesterone and levonorgestrel-releasing injectables has significantly reduced unwanted adolescent pregnancies. Even though the newer methods do not necessarily have better efficacy or safety than the older ones, their ease of use improves compliance among adolescent users. Remarkably, intrauterine devices (IUDs) are currently one of the most extensively used contraceptive techniques in both Europe and America, with approximately 23% of American women aged 15–20 using them—nearly equaling the proportion that uses female sterilization [37]. Despite this development, millions of sexually active adolescents worldwide remain vulnerable to unintended pregnancies and sexually transmitted infections.

### 4.7. Education, Family Planning, and Psychological Aspects

Providing sex education to adolescent girls empowers them with the necessary information to create responsible, respectful relationships. When family planning is included in this education, they become more ready for motherhood and establish mutual respect and trust in sexual relationships—fundamental principles of comprehensive sexual education [38]. The state plays a crucial role by ensuring confidential, supportive environments wherein adolescents can access family planning services. Evidence shows that confidentiality in reproductive healthcare increases service uptake among teens [39]. Yet, millions of teenagers still become pregnant annually, posing significant health risks to both mother and fetus, as teenage pregnancy is associated with higher rates of preeclampsia, eclampsia, dystocia, and iron deficiency anemia, and premature births are more common in pregnant women in this age group [40].

The most effective method of dual protection is still the correct and consistent use of male or female condoms, which simultaneously prevents unwanted pregnancy and sexually transmitted infections (STIs), including HIV [41]. Family planning services therefore need to emphasize correct condom use to adolescents to gain optimum results, including the timeliness of application, the method, and consistency [42].

The theory of “contraceptive psychology” emphasizes how personal and societal determinants, such as personality, cultural norms, and perceived danger, influence contraceptive use [43]. In fact, individuals with a positive attitude towards their sexuality are more likely to utilize effective methods, such as condoms or oral contraceptives, whereas persons with negative attitudes are more likely to use less effective methods, such as withdrawal [44,45,46]. In this study, the exclusive use of IUDs by Christian participants and their complete avoidance among Muslim participants mirror these psychological and cultural mechanisms in practice. Religious interpretations and personal comfort with medical interventions seem to influence both the choice and acceptability of contraception.

### 4.8. Public Health Implications and Concluding Remarks

Although abortion can be the last resort following contraception failure, it has serious health, psychological, and social risks—including possible surgical complications, future infertility, and emotional disturbance [47], highlighting the need for effective contraceptive measures. Typical-use failure rates—representing real-world conditions—vary significantly from perfect-use effectiveness. For example, large-scale statistics report that among 43 nations, male condoms have a typical-use failure rate of 5.4 per 100 women per year, oral contraceptive pills average a rate of 5.5 per 100 women worldwide [45], while withdrawal has an even worse rate of 13.4 [47]. In contrast, long-acting reversible contraceptives (LARCs), such as implants and IUDs, exhibit much lower failure rates (~0.6 and 1.4 per 100 women, respectively) [47].

This difference highlights both the significance of user adherence and the higher reliability of user-independent methods. In addition, emergency contraceptive pills that contain hormones should not be used as a replacement for ongoing combined hormonal contraception because they are less effective. Pregnancy rates are indeed higher with emergency contraception than with the regular use of normal hormonal methods [48].

This study’s findings demonstrate the essential role of family planning clinics in influencing contraception choices while also illustrating the multifaceted influence of religious and sociocultural context. Although religious beliefs appear to influence the choice of EC method, especially regarding IUD use, the general sociocultural environment seems to have a more pronounced impact.

The findings emphasize the need for interventions that strengthen contraceptive education, address interpersonal relationships, and ensure the timely availability of emergency contraception to promote improved reproductive health outcomes.

### 4.9. Limitations

This study has a few limitations that need to be mentioned. First, the cross-sectional, single-center nature of this study restricts the applicability of the findings to the general Greek population, especially in areas with varying cultural or healthcare dynamics. Second, the sample distribution, wherein all IUD users were members of the Christian subgroup, biased comparisons concerning religion and the choice of method. Third, the self-reported nature of the data permitted possible recall and social desirability biases, especially regarding sexual practices and the use of a contraceptive method.

Furthermore, the questionnaire was constructed in-house, and while it was vetted by the hospital’s scientific committee, it was not validated by an external party. Moreover, there were also some wide-based estimates in the regressions, indicating variance and the need for careful interpretation. Lastly, since the study was observational, causal inferences cannot be made; the associations must be interpreted as indicative, not definitive.

Additionally, there were cases of incomplete data. While the overall number of missing-data cases was small, they were not accounted for using imputation or sensitivity analyses, and their exclusion may introduce minor bias or limit the accuracy of the subgroup comparisons.

Some data were incomplete. Although the number of missing cases was small, they were neither imputed nor addressed through sensitivity analyses. Consequently, their exclusion may have introduced minor bias or slightly limited the accuracy of subgroup comparisons.

Despite these limitations, this research provides helpful data about how religious, cultural, and psychological barriers restrict the availability of emergency contraception among young Greek females and can serve as a foundation for larger, multi-site studies.

## 5. Conclusions

This study demonstrates that emergency contraception (EC) use among young women in Northern Greece is influenced by religious, sociocultural, and psychological factors. While Christians and Muslims showed similar contraceptive patterns overall, IUD use occurred exclusively among Christian participants, reflecting cultural and religious differences in method acceptance.

Earlier EC use was more frequent among respondents reporting their male partner’s disapproval or their own lack of information about contraceptives, indicating the role of emotional and interpersonal variables in appropriate EC use. These findings validate the definition of contraceptive psychology, stressing that EC behavior is shaped as much by attitudes and relationship forces as it is by availability.

The poor IUD acceptance and heavy reliance on hormonal pills in Greece mirror long-standing inequalities in education and counseling. Investment in sexual and prescriber education and culture-sensitive reproductive healthcare can strengthen informed decision making and equity-tracked EC access.

## Figures and Tables

**Table 1:** Multinomial logistic regression results.

Comparison	Predictor	*p*-Value	OR	95% CI for OR
12 h vs. 96 h	Living area (rural vs. urban)	0.025	4.23	[1.20, 14.97]
12 h vs. 96 h	Withdrawal (used during first sexual intercourse)	0.023	4.61	[1.24, 17.17]
12 h vs. 96 h	Lack of knowledge (reason for no contraception)	0.017	4.49	[1.30, 15.52]
12 h vs. 96 h	Opposition from partner (reason for no contraception)	0.004	22.82	[2.73, 190.42]
72 h vs. 96 h	Opposition from partner (reason for no contraception)	0.015	4.36	[1.33, 14.25]
72 h vs. 96 h	Lack of knowledge (reason for no contraception)	0.015	2.36	[1.18, 4.73]
72 h vs. 96 h	Other reasons (reasons for no contraception)	0.007	5.03	[1.56, 16.22]

CI: confidence interval; OR: odds ratio.

## Data Availability

The data presented in this study are available upon request from the corresponding author due to privacy.
